# Novel Function of Avian p53 in Binding to ALV-J LTR Contributes to Its Antiviral Roles

**DOI:** 10.1128/mbio.03287-21

**Published:** 2022-01-18

**Authors:** Yueyue Duan, Liyan Cao, Cong Yuan, Xuepeng Suo, Xiangyu Kong, Yulong Gao, Xiangtong Li, Haixue Zheng, Xiaomei Wang, Qi Wang

**Affiliations:** a State Key Laboratory of Veterinary Biotechnology, Harbin Veterinary Research Institute, Chinese Academy of Agricultural Sciences, Harbin, Heilongjiang, China; b State Key Laboratory of Veterinary Etiological Biology, National Foot and Mouth Diseases Reference Laboratory, Lanzhou Veterinary Research Institute, Chinese Academy of Agricultural Sciences, Lanzhou, China; c Institute of Urban Agriculture, Chinese Academy of Agricultural Sciences (CAAS), Chengdu, China; d Chengdu National Agricultural Science and Technology Center, Chengdu, China; University of Calgary

**Keywords:** histone deacetylase, p53, viral replication

## Abstract

Accumulating evidence suggests that p53 is involved in viral infection. However, it remains elusive whether avian p53 orchestrates avian leukosis virus (ALV) replication. We showed that p53 recruits the histone deacetylase 1 and 2 (HDAC1/2) complex to the ALV promoter to shut off ALV's promoter activity and viral replication. HDAC1/2 binding to the ALV promoter was abolished in the absence of p53. Moreover, we collected samples in ALV-infected chickens and found that the acetylation status of ALV-bound H3 and H4 histones correlated with ALV viremia. HDAC inhibitors (HDACi) potently increase ALV replication, but HDACi-promoted viral replication is dramatically reduced in cells with p53 depletion. These data demonstrate that p53 is critical for inhibition ALV replication and suggest that future studies of ALV replication need to account for the potential effects of p53 activity.

## INTRODUCTION

Avian leukosis virus (ALV) has been extensively studied as a tumor retrovirus since it was discovered in 1908 (1). The ALV genome evolves into Rous sarcoma virus (RSV) with cellular *src* (c-*src*) insertion (2). The RSV study was a landmark in the cancer field because RSV's src gene (v-*src*) was the first discovered viral oncogene (3). RSV with v-*src* induces sarcomas in chickens within 1 to 2 weeks (2). ALV does not contain a viral oncogene and is incapable of transforming cells *in vitro*. However, ALV still induces lymphomas for several months because of cellular oncogene regulation by the ALV promoter (4). ALV is a well-characterized system for studying viral promoter sequences in oncogenesis (5, 6). When integrated into the host genome, the retrovirus promoter has regulatory elements that respond to either viral or specialized cellular factors (7). Because ALV does not encode transcriptional factors (8), ALV entirely relies on host transcription factors that bind to the ALV promoter region and drive RNA polymerase II transcription of the provirus (9). The ALV promoter drives high levels of viral and cellular gene transcription in ALV-infected cells. However, the host cell transcription factor involved in ALV promoter activity and viral replication is largely unknown.

The p53 transcription factor has been reported to be involved in the viral life cycle (10). For example, inhibition of p53 activity increases influenza virus infection (11). Overexpression of p53 inhibits HIV-1 long terminal repeat (LTR) transcriptional elongation (12). p53 is capable of directly interacting with viral components to regulate viral replication, such as p53 binding to the SV40 T antigen (13), adenovirus E1B protein (14), and human herpes simplex virus 1 ICP22 protein (15). The p53-mediated transcriptional regulation is divided into transcriptional activation and repression. p53 has been reported to bind to histone acetyltransferases to enhance gene expression (16) or recruit histone deacetylase 1 (HDAC1) to repress the promoter activity (17).

HDACs are a family of enzymes that catalyze the deacetylation of acetylated protein, consequently influencing gene expression and chromatin remodeling (18). In general, high HDAC activity is associated with condensed, transcriptionally inactive chromatin. HDAC family members 1, 2, and 3 could form a large nuclear complex that is crucial for transcriptional repression and epigenetic modifications. HDAC1 and HDAC2 are the best characterized of the HDACs and are generally found in nuclear complex and are then recruited by DNA binding proteins (19). Here, we showed that p53 directly binds with the ALV promoter and recruits the HDAC1/2 complex to inhibit ALV promoter activity by reducing histone acetylation at the ALV promoter. These findings indicate that p53 is crucial for the regulation of ALV replication and might shed light on ALV-induced tumorigenesis.

## RESULTS

### Avian p53 and the HDAC complex are required for silencing of ALV promoters.

**FIG 1:**
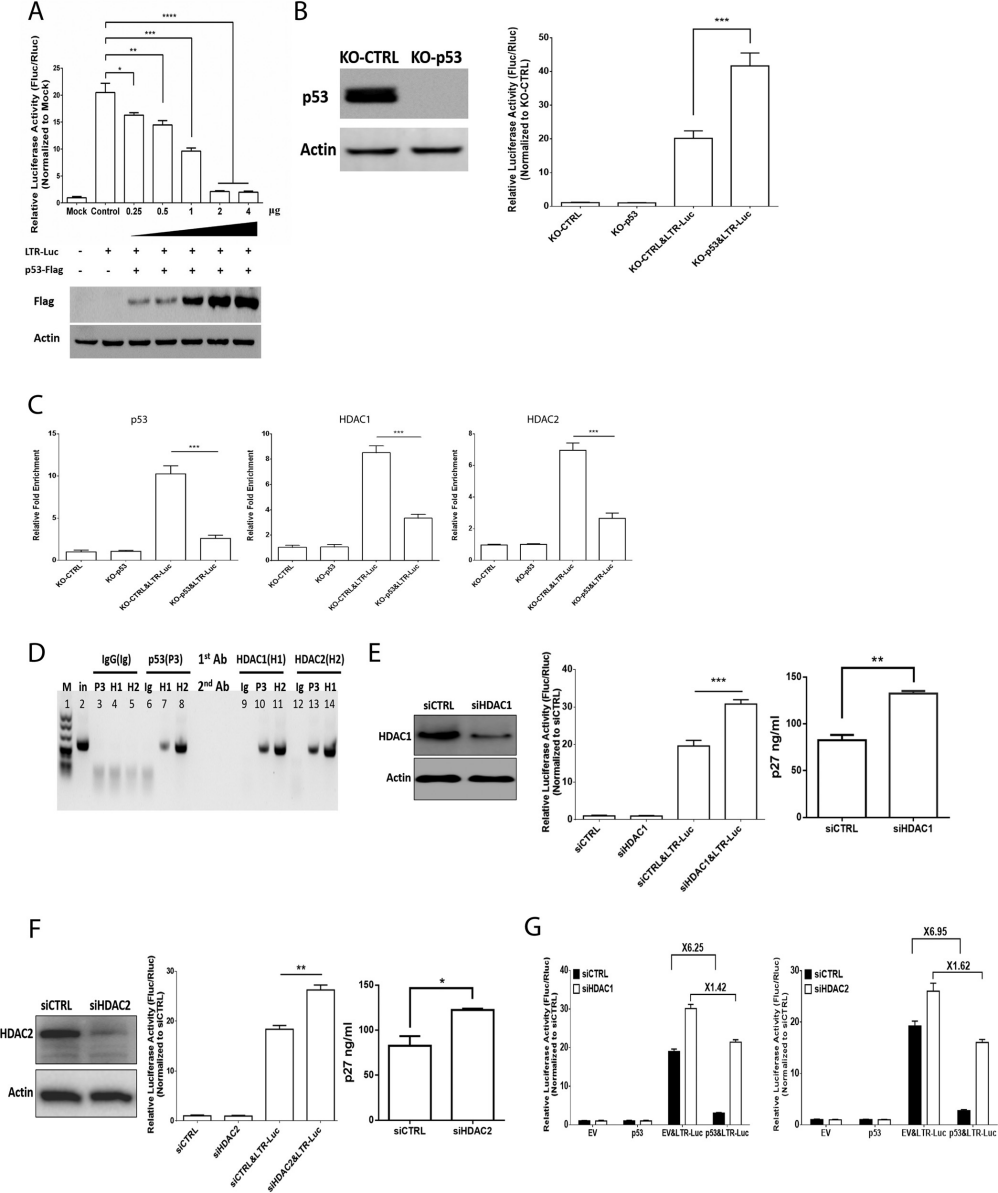
p53 inhibits ALV-J promoter activity. (A) p53 overexpression inhibits ALV-J LTR promoter activity in a dose-dependent manner. (B) ALV-J LTR promoter activity was increased in DF-1 cells with depletion of endogenous p53. (C) Depletion of endogenous p53 prevents recruitment of HDAC1 and HDAC2 to the ALV-J promoter. DF-1 cells were transfected with ALV-J LTR-mediated reporter followed by ChIP assays. Each ChIP DNA fraction's threshold cycle (*C_T_*) value was normalized to the IgG DNA fraction's *C_T_* value (ΔΔ*CT*) at the same time point. IgG is defined as 1. Error bars stem from three technical replicates; *n* = 3. (D) Re-ChIP PCR assay of p53-HDAC1/2 interaction at ALV-J LTR promoter in DF-1 cells after ALV-J LTR-mediated reporter transfection. First-round ChIP (1st antibody [Ab]) against p53 (P3), HDAC1 (H1), HDAC2 (H2), or IgG (Ig). Eluted samples were subject to re-ChIP (2nd antibody). Lane 2 was input (in). (E, Left) siRNA-mediated knockdown of HDAC1 in DF-1 cells was performed and confirmed by Western blot analysis. (E, Middle) DF-1 cells were then transfected with an ALV-J LTR-mediated reporter. (E, Right) ALV-J infection and replication in control and siRNA-mediated knockdown of HDAC1 in DF-1 cells. ALV-J replication was measured on day 6 postinfection by p27 ELISA. Error bars represent ± SD for triplicate experiments. (F, Left) siRNA-mediated knockdown of HDAC2 in DF-1 cells was performed and confirmed by Western blot analysis. (F, Middle) DF-1 cells were then transfected with an ALV-J LTR-mediated reporter. (F, Right) ALV-J infection and replication in control and siRNA-mediated knockdown of HDAC2 in DF-1 cells. ALV-J replication was measured on day 6 postinfection by p27 ELISA. Error bars represent ± SD for triplicate experiments. (G) p53 and ALV-J LTR-mediated reporter overexpression in siRNA-mediated knockdown of HDAC1 or HDAC2 in DF-1 cells. Thirty-six hours after transfection, cells were harvested and lysed for assessment of luciferase activity.

p53 has been reported to inhibit HIV-1 LTR promoter activity (20). To explore whether avian p53 could affect ALV LTR promoter activity, we took cotransfection ALV LTR-mediated luciferase reporter plasmid with plasmid expressing p53. As shown in Fig. 1A, p53 inhibits ALV LTR promoter transcription activity in a dose-dependent manner. ALV LTR promoter transcription activity was dramatically increased in DF-1 cells with depletion of endogenous p53 (Fig. 1B). Thus, we investigated the roles of p53 for ALV LTR transcription repression. Chromatin immunoprecipitation (ChIP) analysis showed that the binding of p53 and HDAC1/2 to ALV LTR occurs during overexpression of ALV LTR-mediated luciferase reporter plasmid (Fig. 1C). The ALV LTR displays reduced binding of HDAC1 and HDAC2 in DF-1 cells with depletion of endogenous p53, which indicates that HDAC1 and HDAC2 bind to ALV LTR that requires p53 expression (Fig. 1C). To further reveal the recruitment of HDAC1/2 to p53 promoter by p53, we performed sequential ChIP (ChIP-re-ChIP) and found that p53 formed a complex with HDAC1/2 on ALV LTR (Fig. 1D). Silencing of HDAC1/2 increased ALV LTR-mediated luciferase activity and ALV-J replication (Fig. 1E and F). Moreover, silencing of HDAC1/2 attenuated p53-mediated repressive effect on ALV LTR promoter activity (Fig. 1G).

### Avian p53 inhibits ALV replication through binding to the ALV promoter.

**FIG 2:**
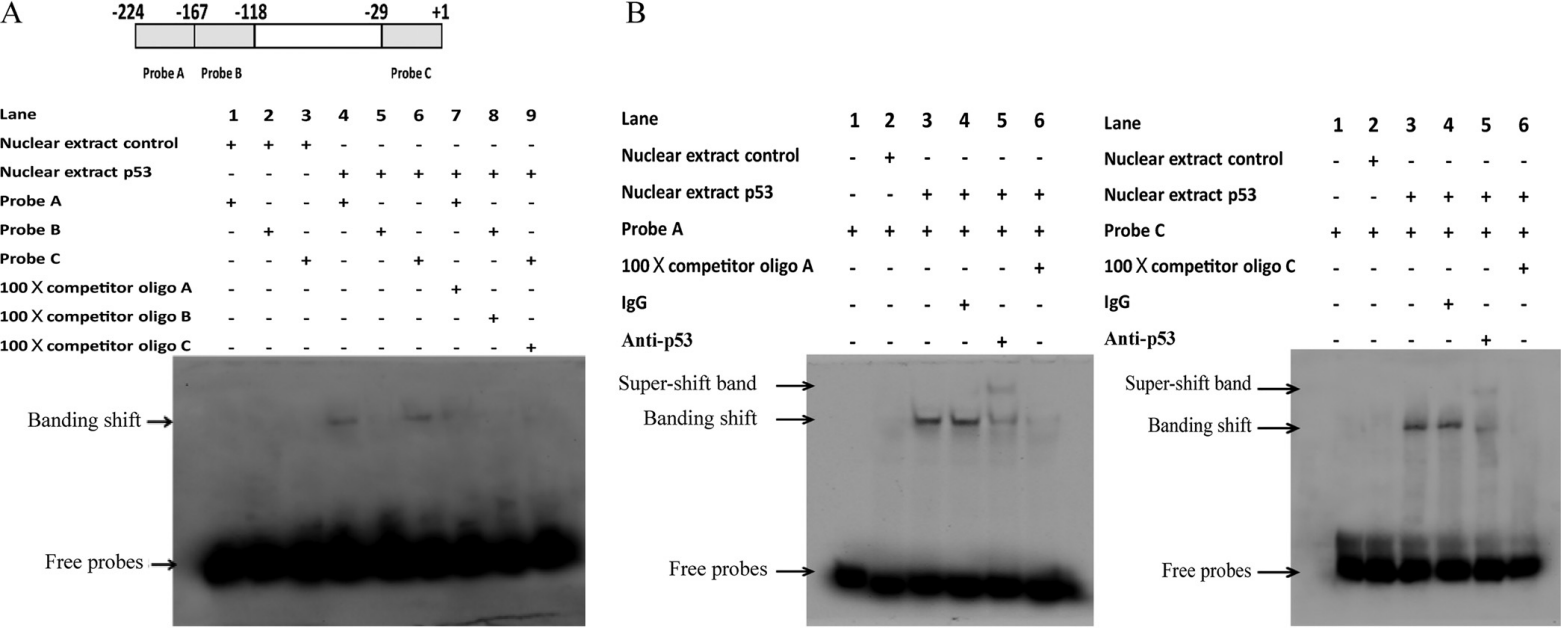
p53 binds to ALV-J LTR. (A, Top) Diagram of ALV-J long terminal repeat (LTR) and DNA fragments used in electrophoretic mobility shift assay (EMSA). The shaded boxes indicate the EMSA probe-corresponding fragment. The size and location of DNA fragments used in the EMSA are indicated. (A, Bottom) EMSA of *in vitro* binding of p53 to probes A and C but not probe B corresponding to the LTR region. Nuclear extract from Hep3B cells transfected with a plasmid expressing avian p53 protein (NEP53) was used for EMSA and band shift of protein-DNA was abolished with excessive nonlabeled probe (NLP). Nuclear extract from Hep3B cells transfected with empty plasmid was the negative control (NEC). Lane 1, NEC with probe A; lane 2, NEC with probe B; lane 3, NEC with probe C; lane 4, NEP53 with probe A; lane 5, NEP53 with probe B; lane 6, NEP53 with probe C; lane 7, NEP53 with probe A and NLP corresponding to probe A; lane 8, NEP53 with probe B and NLP corresponding to probe B; lane 9, NEP53 with probe B and NLP corresponding to probe C. (B, Left) EMSA of binding of avian p53 to probe A region in ALV-J LTR. Lane 1, probe A; lane 2, NEC with probe A; lane 3, NEP53 with probe A; lane 4, supershift with IgG in NEP53 with probe A; lane 5, supershift with p53 antibody (PAb 240); lane 6, NEP53 with probe A and NLP corresponding to probe A. (B, Right) EMSA of avian p53 binding to probe C region in ALV-J LTR. Lane 1, probe C; lane 2, NEC with probe C; lane 3, NEP53 with probe C; lane 4, supershift band with IgG in NEP53 with probe C; lane 5, supershift with p53 antibody (PAb 240); lane 6, NEP53 with probe C and NLP corresponding to probe C.

To further explore how p53 binds to ALV-J LTR, we performed electrophoretic mobility shift analysis (EMSA) to confirm the binding site of p53 on ALV-J LTR. Two different probes targeting ALV LTR were used to check the p53 binding status of nuclear extracts prepared from cells expressing p53. As shown in Fig. 2A and B, the binding of avian p53 was found to be specific, and the DNA-protein complexes were especially abolished in the presence of a 100-fold competitor oligonucleotide or antibody against p53. All of these indicate that avian p53 specifically binds to the ALV-J LTR region.

**FIG 3:**
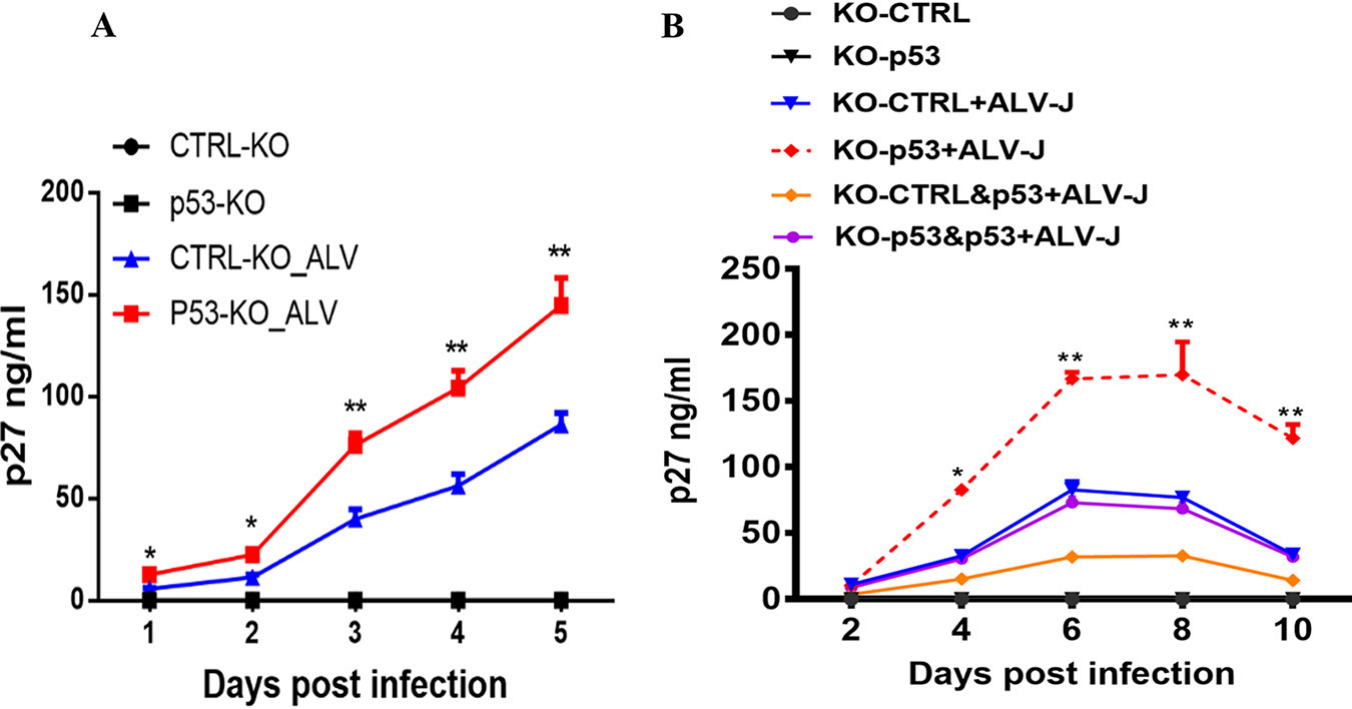
p53 inhibits ALV-J replication. (A) ALV-J infection and replication in control and p53 knockout CEF cells. ALV-J replication was measured over 5 days by p27 ELISA. Error bars represent ± SD for triplicate experiments. (B) ALV-J infection and replication in control and p53 knockout DF-1 cells. Reexpression of p53 in control and p53 knockout DF-1 cells. ALV-J replication was measured over 10 days by p27 ELISA. Error bars represent ± SD for triplicate experiments.

To test the importance of avian p53 inhibition on ALV promoters, we explored ALV-J replication by testing primary chicken embryo fibroblasts (CEF) with depletion of p53. As shown in Fig. 3A, ALV-J exhibited a dramatic enhancement of virus replication in p53 knockout (KO) CEF cells over time, determined by the p27 enzyme-linked immunosorbent assay (ELISA) (Fig. 3A). We next tested ALV-J replication in p53 knockout DF-1 cell lines, and the knockout cell lines were reconstituted with p53. As shown in Fig. 3B, the knockout of p53 substantially accelerates ALV replication, and viral replication is restored when p53 knockout cells are reconstituted with avian p53. All of these indicate that p53 directly inhibits ALV-J replication.

### The acetylation status of ALV-bound H3 and H4 histone correlates with ALV viremia level *in vivo*.

**FIG 4:**
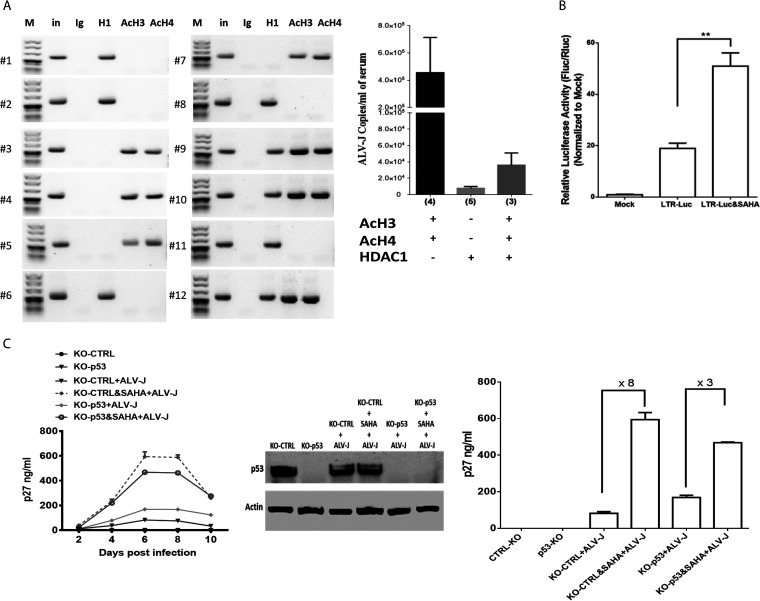
Acetylation status of ALV-J genome-bound H3 and H4 histones correlates with ALV-J viremia levels in chickens in clinical settings. (A, Left) ChIP of liver nuclear extracts from 6 ALV-J-positive chickens with ALV-J infection by using specific antibodies to AcH3, AcH4, HDAC1, or control IgG, and specific PCR primes for ALV-J promoter. (A, Right) ALV-J quantification in the serum samples from 12 ALV-J-positive chickens with active (AcH3-AcH4 positive/HDAC1 negative, 4 cases; AcH3-AcH4 positive/HDAC1 positive, 3 cases) or suppressed (AcH3-AcH4 negative/HDAC1 positive, 5 cases) ALV-J replication was performed by ALV-J proviral genome copies quantitative analysis by quantitative PCR (qPCR). (B) ALV-J LTR-mediated reporter overexpression in DF-1 cells with 1 μM SAHA treatment. Thirty-six hours after transfection, cells were harvested and lysed for assessment of luciferase activity. (C, Left) ALV-J infection and replication in control and p53 knockout DF-1 cells with SAHA treatment. ALV-J replication was measured over 10 days by p27 ELISA. Error bars represent ± SD for triplicate experiments. (C, Middle) p53 expression level in each group was confirmed by Western blot analysis. (C, Right) p27 levels in the supernatant at 6 days postinfection (dpi) were analyzed to determine the SAHA-mediated regulation fold on ALV-J replication in control and p53 knockout DF-1 cells.

The p27 protein level in serum is commonly used as a surrogate marker of ALV replication in chickens in clinical settings. To assess whether the acetylation status of ALV LTR-bound histones affects ALV replication *in vivo*, we have applied the same ChIP/ALV-specific PCR approach to spleen obtained from ALV-infected chickens. We found a striking correlation between ALV LTR-bound H3 and H4 acetylation and ALV viremia levels (Fig. 4A). These findings suggest that histone deacetylase inhibitors (HDACi) such as suberoylanilide hydroxamic acid (SAHA), already approved by the United States Food and Drug Administration (FDA), might modulate ALV replication. To identify our hypothesis, we investigated SAHA's role in ALV LTR promoter activity and ALV replication. As shown in Fig. 4B, 1 μM SAHA treatment dramatically increased ALV LTR promoter activity in DF-1 cells. Moreover, SAHA dramatically enhances ALV replication ∼8-fold. However, SAHA-mediated ALV replication enhancement is ∼3-fold in cells’ depletion of p53, consistent with the above results that p53 recruited HDAC1/2 in ALV LTR (Fig. 4C). We also tested the viability of DF-1 cells affected by SAHA, which showed that SAHA decreased DF-1 cell viability in a dose-dependent manner (Fig. S1A in the supplemental material). We used 1 μM SAHA to explore its function in ALV replication, which does not affect DF-1 cells’ viability.

10.1128/mbio.03287-21.1 FIG S1 SAHA affects the viability of DF-1 cells. Cell viability was assessed using the 3-(4,5-dimethyl-2-thiazolyl)-2,5-diphenyl-2H-tetrazolium bromide (MTT) test. A dose-dependent decrease of DF-1 cell viability was observed. Download FIG S1, TIF file, 0.3 MB. Copyright © 2022 Duan et al. 2022 Duan et al. https://creativecommons.org/licenses/by/4.0/ This content is distributed under the terms of the Creative Commons Attribution 4.0 International license.

## DISCUSSION

ALV-J is dependent on its host cell for viral replication. Antiviral restriction factors are the cellular proteins that constitute a defense line of blocking viral replication and propagation. Previous studies have indicated that p53 plays a role in inhibiting viral replication of several viruses, including influenza viruses (21), HIV-1 (20), and SARS-CoV (22). However, the role of avian p53 in ALV-J infection is not fully understood. Our study demonstrates that p53 inhibits ALV-J replication by recruiting HDAC1 and HDAC2 on the ALV-J promoter. Moreover, using the ChIP assay, we found that ALV-J replication is associated with histones H3 and H4 acetylation onto the ALV-J promoter. The increased fold of histone deacetylase inhibitors (SAHA) on ALV-J replication was dramatically decreased in p53 knockout cells, which suggests that histones H3 and H4 acetylation on ALV-J requires p53 expression.

Previous studies have shown that HDAC1/2 remodeling acetylation/deacetylation events regulate promoter activity during viral replication, including HIV-1, herpes simplex virus (HSV), Kaposi’s sarcoma-associated herpesvirus (KSHV), and Epstein-Barr virus (EBV). p53 has been reported to interact with HDAC1 and HDAC2 (22). In our study, we showed that p53 inhibits ALV-J LTR activity, requiring HDAC1 and HDAC2. EMSA, ChIP, and re-ChIP assays showed that p53, HDAC1, and HDAC2 are complex ALV-J promoters. HDAC1 and HDAC2 are the components of corepressor complexes that could switch off gene expression via binding on the gene promoter (23). SAHA (vorinostat) is a potent inhibitor of class I and class II HDACs that belongs to the group of hydroxamic acids. SAHA has been reported to stimulate HIV replication from latently infected cells and hepatitis B HBV replication (24, 25). Here, SAHA-mediated ALV replication enhancement is dramatically reduced in cell depletion of p53, which suggests p53 is the key factor for recruiting HDAC1 and HDAC2 to modulate ALV replication.

In summary, we showed that p53 is a negative regulator in ALV-J replication by recruiting HDAC1 and HDAC2 *in vitro* and *in vivo*. Understanding the role of p53 in ALV-J replication may shed light on the natural protection occurring in ALV-J infection.

## MATERIALS AND METHODS

### Cells and viruses.

DF-1 cells (ATCC; CRL-12203) were grown in Dulbecco's modified Eagle's medium (DMEM; Invitrogen, Shanghai, China) supplemented with 10% fetal bovine serum, 100 μg/mL penicillin, and streptomycin. The HPRS-103 prototype virus was a generous gift from Venugopal Nair (Avian Viral Diseases Program, BBSRC Institute for Animal Health, Compton, UK).

### Luciferase reporter assays.

To assess luciferase reporter activity, including the activity of ALV LTR-Luc, a dual‐luciferase reporter assay was performed as described in our previous studies (26). In brief, DF-1 cells were seeded in 48-well plates. Twelve hours later, cells were transfected with the ALV LTR-mediated luciferase reporter plasmid. The pRL-TK Renilla luciferase reporter plasmid (Promega, USA) was cotransfected for normalization of the transfection efficiency and was the internal control. Thirty-six hours after transfection, cells were harvested and lysed for assessment of luciferase activity with a dual-luciferase reporter assay kit (Vazyme, China) according to the manufacturer’s protocol. Luciferase activity was measured in a Centro XS3 LB 960 microplate luminometer (Berthold Technologies, Germany). Relative luciferase activity was calculated by normalizing firefly luciferase activity to Renilla luciferase activity.

### Antibodies and cell transfection.

Transfection of DF-1 cells was performed using Lipofectamine 2000 (Invitrogen) following the manufacturer's protocol. For p53 detection, antigen detection was performed with an anti-p53 antibody (Novus Biologicals). The anti-HDAC1 antibody was purchased from OriGene Technologies. The anti-HDAC2 antibody was purchased from GeneTex.

### RNAi and CRISPR-Cas9.

HDAC1-targeting small interfering RNA (siRNA) sequences designed by siRNA design tools from GenScript were 5′-CTACGGCCTT TACAGGAAG-3′, 5′-GCCGGTGATATCTAAAGTG-3′, and 5′-TCTGACCATC AAAGGTCAT-3′. HDAC2-targeting siRNA sequences designed by siRNA design tools from GenScript were 5′-CCAATGAGTTGCCATATAA-3′, 5′-CAATTGGGCTGGAGGACTA-3′, and 5′-ACAGGAGACTTGAGGGATA-3′. siRNAs were delivered to cells using Lipofectamine RNAiMax reagent (Life Technologies) following the manufacturer's guidelines. Briefly, siRNA oligonucleotides and RNAiMax were separately diluted in Opti-MEM medium (Life Technologies) and then mixed and incubated for 20 to 30 min at room temperature. Subsequently, the siRNA-RNAiMax complex was mixed with the cells and transferred to a cell culture plate. Cells were incubated at 37°C for 72 h before infecting or further processing them.

CRISPR-Cas9 lentiviral vector lentiCRISPR v2 was purchased from Addgene. The guide RNA (gRNA) sequence used for targeting p53 is CACCGGAATAAGGTCTATTGCCGCC. Lentiviruses for CRISPR were produced by CaCl2-BES transfection of 293T cells (10-cm plate) using 10 mg vector, 15 mg pMDLg/pRRE, 4 mg pRSV-Rev, and 5 mg pCMV-VSV-G. One million DF-1 cells were transduced with lentiviruses and, after 3 days, selected with 2 mg/mL puromycin for 7 days.

### ChIP and EMSA.

According to the manufacturer's instructions, ChIP assays were conducted with Simple ChIP kits (Cell Signaling Technology, Beverly, MA, USA). Primers for the ALV-J promoter were forward, 5′-CGGGGTACCTGTAGTGTTATGCAATACTC-3′, and reverse, 5′-CCGCTCGAGGTTTATTGTGTCGGGCTAGG-3′. Nuclear extracts were prepared from Hep3B cells transfected with a plasmid expressing avian p53 or empty plasmid. EMSA was performed using nonradioactive EMSA kits following the manufacturer's instructions (Pierce Biotechnology, Rockford, IL). Probe A was 5′-AAGAAAAAGGCACTGTACACGTCGATTGGTGGAAGTAAGGTGGTATGATC-3′, and probe C was 5′-TCCGCATTACAGAGATATTGTATTTAAGTGCCTAGCCCGACACAATAAAC-3′. A 5′-end oligonucleotide was labeled with biotin. For competition experiments, 100-fold excess unlabeled, double-stranded oligonucleotides were added; for supershift experiments, 1 mg p53 antibody was added.

### ALV-J infection and detection.

The titer of ALV-J was determined by infecting a known number of DF-1 cells with the titrated amount (p27). The supernatant of ALV-J P27 group-specific antigen was detected using a subgroup J ALV antibody test kit (Idexx, China) at each time point, respectively. The assay was conducted according to the manufacturer's instructions. Each sample's absorbance (optical density [OD]) was measured with a Bio-Tek Instruments EL310 microplate autoreader at a wavelength of 450 nm. At the same time, total RNA was extracted from cells with RNeasy minikits (Omega, USA) according to the manufacturer's instructions. According to the manufacturer's instructions, first-strand cDNA synthesis kits were used (Invitrogen, USA) for reverse transcription. Real-time RCR was performed to detect the viral load. Clinical samples were collected from laying hens at the age of 22 weeks.

### Statistical analysis.

All statistical analyses were performed with unpaired Student's *t* test and were considered significant when *P* values were less than 0.05. *, *P < *0.05; **, *P < *0.01; ***, *P < *0.001.
