# Supplementary figures and images for: Novel Function of Avian p53 in Binding to ALV-J LTR Contributes to Its Antiviral Roles
Source: mBio. 2022 Jan 18;13(1):e03287-21. doi: 10.1128/mbio.03287-21 (PMC8764537; doi:10.1128/mbio.03287-21)

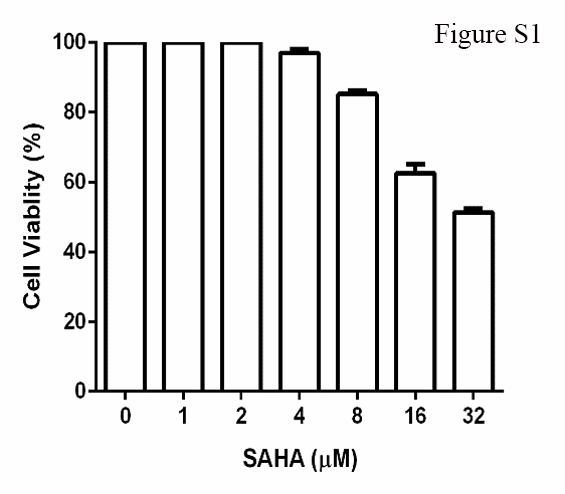

Supplement: FIG S1 [file mbio.03287-21-sf001.tif]
